# Comprehensive Strategies for Metabolic Syndrome: How Nutrition, Dietary Polyphenols, Physical Activity, and Lifestyle Modifications Address Diabesity, Cardiovascular Diseases, and Neurodegenerative Conditions

**DOI:** 10.3390/metabo14060327

**Published:** 2024-06-11

**Authors:** Giovanni Martemucci, Mohamad Khalil, Alessio Di Luca, Hala Abdallah, Angela Gabriella D’Alessandro

**Affiliations:** 1 University of Bari Aldo Moro, 70126 Bari, Italy; gmartem@libero.it; 2 Clinica Medica “A. Murri”, Department of Precision and Regenerative Medicine and Ionian Area (DiMePre-J), University of Bari Medical School, 70121 Bari, Italy; halaabdallah18@gmail.com; 3 Department of Soil, Plant and Food Sciences, University of Bari Aldo Moro, 70126 Bari, Italy; alessio.diluca@uniba.it (A.D.L.); angelagabriella.dalessandro@uniba.it (A.G.D.)

**Keywords:** metabolic syndrome, diabesity, microbiota, dietary polyphenols, physical activity

## Abstract

Several hallmarks of metabolic syndrome, such as dysregulation in the glucose and lipid metabolism, endothelial dysfunction, insulin resistance, low-to-medium systemic inflammation, and intestinal microbiota dysbiosis, represent a pathological bridge between metabolic syndrome and diabesity, cardiovascular, and neurodegenerative disorders. This review aims to highlight some therapeutic strategies against metabolic syndrome involving integrative approaches to improve lifestyle and daily diet. The beneficial effects of foods containing antioxidant polyphenols, intestinal microbiota control, and physical activity were also considered. We comprehensively examined a large body of published articles involving basic, animal, and human studie, as well as recent guidelines. As a result, dietary polyphenols from natural plant-based antioxidants and adherence to the Mediterranean diet, along with physical exercise, are promising complementary therapies to delay or prevent the onset of metabolic syndrome and counteract diabesity and cardiovascular diseases, as well as to protect against neurodegenerative disorders and cognitive decline. Modulation of the intestinal microbiota reduces the risks associated with MS, improves diabetes and cardiovascular diseases (CVD), and exerts neuroprotective action. Despite several studies, the estimation of dietary polyphenol intake is inconclusive and requires further evidence. Lifestyle interventions involving physical activity and reduced calorie intake can improve metabolic outcomes.

## 1. Introduction

Metabolic syndrome (MS) affects approximately 30% of the world’s population, and its prevalence is on the rise in Western countries due to factors such as sedentary lifestyles, diets, and environmental influences. In European countries, 26% of the population suffers from this disease [1]. The incidence of MS tends to increase with age [2], especially among women [3]. MS can be defined as a cluster of multifactorial biochemical, physiological, clinical, and metabolic disorders leading to obesity, diabetes, cardiovascular, and cerebrovascular diseases. These disorders are linked to altered metabolism, such as dyslipidemia, hyperglycemia, impaired glucose tolerance related to hyperinsulinemia and insulin resistance, and a low baseline degree of inflammation [4,5,6,7].

All free radicals are involved in body pathophysiological processes [8]. Superoxide, a highly reactive free radical, can cause damage to cellular molecules (DNA, proteins, and lipids) and impair the functionality of organs, leading to disease [9]. Hydroxyl radicals are implicated in several disorders, including cardiovascular disease (CVD) and cancer [10]. Nitric oxide is involved in many physiological processes, such as vasodilation and blood pressure regulation, as well as pathological processes such as neurodegenerative disorders and heart diseases [11]. The imbalance between the generation of free radicals/oxidants and antioxidant defenses leads to oxidative stress, disruption of redox signaling, and molecular damage, contributing to various diseases [8,12]. Many studies suggest a strong association between high oxidative stress and MS [13,14,15] and its related conditions such as obesity [15,16], diabetes [17], cardiovascular and neurodegenerative diseases [15,18], as well as aging processes [12]. Antioxidant systems, including endogenous antioxidant defense mechanisms along with exogenous antioxidants such as polyphenols from diet sources, act by blocking the formation of free radicals or interrupting the propagation of the free radical chain reaction [19]. To prevent the development of metabolic disorders, it is important to involve the investigation of natural plant-based antioxidants, represented mainly by polyphenols. Polyphenols, most present in plant-based diets, spices, fruit, vegetables, and cereals, may reduce the risk of MS [20]. Several studies reported the beneficial effects of polyphenols in the prevention of diabesity, cardiovascular, and neurodegenerative diseases [21,22,23,24].

A sedentary lifestyle and malnutrition play a key role in MS and related diseases. Overweight and obesity result from a chronic imbalance between energy intake and energy expenditure. A diet rich in fat and carbohydrates can lead to obesity and chronic inflammation through oxidative stress and the suppression of the antioxidant system [25]. A high-fat diet has been associated with insulin resistance [26]. Additionally, there is a strong correlation between obesity and insulin resistance mediated through leptins and high levels of free fatty acids [27,28] derived from meals and lipolysis of adipose tissue. Abdominal obesity, being metabolically more active, exhibits increased sensitivity to lipolysis stimulation and a reduced insulin response [29,30]. Excessive insulin secretion leads to beta-cell dysfunction [31]. β-cell dysfunction, defective proliferation, and growth lead to type-2 diabetes [32]. Insulin resistance can induce significant alterations in the compensatory responses of insulin secretion, resulting in decreased glucose tolerance. This, in turn, can lead to the inhibition of insulin activity and secretion, accelerating the onset of type 2 diabetes.

Increased inflammation, brought about by the alteration of redox signaling pathways, gene expression of inflammatory cytokines, chemokines, and growth factors, can lead to insulin resistance, diabetes, and cardiovascular damage [14]. This occurs through altered cellular and nuclear mechanisms, including impaired DNA damage repair and cell cycle regulation [33]. Individuals with obesity face a higher risk of developing colon cancer, oesophageal adenocarcinoma, and cholangiocarcinoma [34], while diabetes is identified as a predictor of mortality related to colon, pancreatic, breast, liver, and bladder cancer [35].

Diabetes is recognized as the primary risk factor for CVD, encompassing both macrovascular and microvascular conditions, along with hypertension characterized by endothelial dysfunction [36,37].

CVD is associated with metabolic disorders arising from diabetes and dyslipidemia [38,39]. Increased cardiac lipid accumulation and altered metabolism in obesity result in cardiovascular complications, including reduced systolic function due to the deposition of myocardial triacylglycerol and left ventricular hypertrophy [40]. Adiponectin, a cytokine derived from adipose tissue, is important in metabolic disorders leading to cardiac death [41]. Patients with obesity/diabetes often exhibit low levels of adiponectin, contributing to elevated LDL and decreased HDL levels. Reduced adiponectin levels are associated with left ventricular hypertrophy, especially in patients with diabetes and obesity [42].

Several studies indicate that the gut microbiota is involved in developing obesity, diabetes, and associated comorbidities [43]. The gut microbiota plays an important role in the development and progression of metabolic dysfunction linked to MS and its prevention. The symbiotic relationship with the host ensures adequate development of the metabolic system, performing important functions in health such as maintaining nutritional status and supporting immunity [44,45,46]. Dysbiosis has been observed in children [47] obese individuals [48], and obese women with MS [49].

Neurodegenerative disorders result from various pathological mechanisms, including oxidative stress, mitochondrial dysfunction, neuroinflammation, dysfunction in protein metabolism and proteasomes, and the formation of advanced glycation end products [50]. Mitochondrial dysfunction has been associated with axonal degeneration and the impairment of the viability of oligodendrocytes [51]. Inflammation and immune system alterations have also been linked to neurodegenerative disorders [52]. Proinflammatory cytokines and other inflammatory mediators, such as prostaglandins and complement factors, favor the recruitment of peripheral immune cells, promoting neuroinflammation. Alzheimer’s [53,54] and Parkinson’s diseases [55] are considered the most common neurodegenerative disorders.

Lifestyle changes, including diet modification along with regular physical activity, can be considered to have a pivotal role in the prevention and treatment of MS, CVD, and neurodegenerative disorders. This review outlines the relationship between nutrition, gut microbiota, antioxidants, and physical activity in managing metabolic syndrome and related cardiovascular and neurodegenerative diseases. Possible intervention strategies for prevention and treatment through diet, antioxidant polyphenols, and exercise will be discussed.

### Literature Search Methodology

The literature search was conducted using a variety of databases to ensure comprehensive coverage. These included PubMed, Google Scholar, Web of Science, and Scopus. A combination of specific and broad keywords related to the study was used to capture a wide range of relevant studies. Keywords included “metabolic syndrome”, “nutrition”, “Mediterranean diet”, “polyphenols”, “physical activity”, “diabesity”, “cardiovascular diseases”, and “neurodegenerative diseases”. Articles were selected based on their relevance to the interaction between nutrition, physical activity, and the management of metabolic syndrome. Emphasis was placed on peer-reviewed articles that contribute significantly to the understanding of the topic. Non-peer-reviewed articles, articles not available in English, and those with limited accessibility were excluded to maintain the quality and relevance of the references. Special attention was given to identifying human studies that highlight the synergistic effects of nutrition and physical activity on metabolic syndrome and its associated conditions. The review focused on studies that provided insights into mechanisms, intervention strategies, and long-term outcomes.

## 2. Therapeutic Strategies against Metabolic Disorders

A sedentary lifestyle characterized by a lack of physical activity combined with the consumption of a Western diet high in energy, cholesterol, saturated fatty acids, animal protein, salt, and low in fiber contributes significantly to the development of MS [56,57]. Diabesity and its complications, such as CVD, can manifest from childhood and adolescence to early adulthood [58]. The adoption of an appropriate diet and lifestyle could hinder the development of metabolic disorders in children and adolescents. For obesity, other therapeutic options (pharmacotherapy, bariatric surgery) are either not available or not recommended [59,60,61]. Modern approaches to managing the risk factors involved in MS suggest lifestyle changes, including modifications to diet and physical activity [4,62] and the use of drugs involved in nutrient metabolism [63]. However, medications are often expensive, monotherapeutic, with poor patient compliance, and implicated with side effects during prolonged use [62,64]. Thus, alternative methods for managing metabolic dysfunctions focus on lifestyle changes through a multidisciplinary approach, such as controlling body weight, maintaining a healthy diet, and engaging in physical activity, which have shown promising results in conditions of MS [65,66].

### 2.1. Nutrition Strategies against Diabesity

#### 2.1.1. Nutrition Management of Diabesity

There is a close relationship between obesity and diabetes [15,67,68] defined as “diabesity”. Diabesity involves both genetic and environmental factors and manifests as a connection between two metabolic disorders characterized by defects in cellular insulin, whose sensibility is primarily attributed to insulin resistance and insulin deficiency [15,69].

Lifestyle is important in the prevention and treatment of obesity, diabetes, and diseases linked to MS. Obesity is characterized by a body mass index (BMI) greater than 30 kg/m^2^, while a BMI between 25 and 30 kg/m^2^ is classified as overweight [70]. Weight loss can be achieved through various means, including dietary interventions, exercise, and bariatric surgery. Bariatric surgery is suggested for individuals with a BMI greater than 35 kg/m^2^ who have concurrent risk factors such as diabetes and may lead to partial remission of diabetes when combined with adequate lifelong care [71]. Clinical studies have shown that reducing weight can improve cardiovascular risk associated with obesity and diabetes [72,73,74]. The most significant effects, which regard the reduction of visceral adiposity and adipocyte size [75,76], are observed with a weight reduction of at least 10%. 

Weight loss may prevent and improve type 2 diabetes (T2D) in people with prediabetes, and it has the potential to reverse T2D. Lifestyle interventions have been shown to reduce the conversion of prediabetes to T2D by 58% [77]. For overweight or obese people with T2D, a weight loss of at least 5% is recommended through a combination of diet, physical activity, and behavioral therapy [78]. Greater weight loss can lead to reversing metabolic abnormalities associated with T2D, resulting in improved blood sugar levels and even diabetes remission [79,80,81]. Body weight loss has positive effects on blood glucose control, insulin sensitivity, and comorbidities [82]. A 10 kg weight reduction has been shown to improve glycemia, address diabetes comorbidities (hypertension, fatty liver disease, depression, and obstructive sleep apnea syndrome), and reduce overall mortality by 25% in people with T2D [83]. People with obesity and T2D are advised to maintain their weight within the BMI range of 18.5–24.9 kg/m^2^ [84].

Waist circumference appears to be a more accurate predictor of cardiovascular risk than BMI [85]. Women with a waist circumference of ≥ 88 cm and men with ≥102 cm are recommended to reduce body weight. For people with diabetes and a BMI ≥ 35 kg/m^2^, weight reduction should be at least 10% [85]. It is important that BMI may not always reflect increases in adiposity or the distribution of body fat, which is a more reliable predictor of cardiometabolic complications [86,87]. Additionally, certain ethnic groups may experience complications associated with increased adiposity at a lower BMI [88,89]. Adopting a diet low in fat, refined carbohydrates, and salt, along with reduced caloric intake and cholesterol, saturated fat, and increased consumption of unsaturated fat, complex carbohydrates, and fiber, has been shown to improve risk factors associated with MS, diabesity, and CVD [66,90,91].

Calorie restriction in the diet promotes weight loss and is associated with improvements in lipid and cytokine profiles, potentially reducing cardiovascular risks [92]. Dietary restriction can improve gut dysbiosis associated with obesity and diabetes, making it a potential therapeutic approach to prevent or treat these metabolic disorders [93]. Dietary energy restriction (20–40%) has been shown to improve health conditions and reduce the risks of metabolic disorders [94,95]. Additionally, dietary protein restriction has been associated with a reduction in the risk of diabetes and cancer [94]. Notably, protein restriction appears to yield similar clinical results as calorie restriction without reducing calorie intake [96].

Short-term calorie restriction is associated with 5–10% weight loss, but long-term compliance poses a significant challenge due to the tendency to regain lost weight [97,98,99]. Consequently, alternative dietary strategies have been explored to manage energy homeostasis and obesity. Intermittent fasting, for instance, has been shown to result in comparable or greater weight loss and improved metabolic status compared to continuous calorie restriction [100,101,102,103]. Time-restricted feeding involves limiting dietary energy intake to a window of 4 to 12 h by extending the fasted state without altering caloric intake [104]. A specific regimen of time-restricted feeding, comprising 8 to 9 h a day for 5 days with *ad libitum* intake for 2 days, has been demonstrated to reverse or limit diet-induced obesity [105,106].

The implementation of strategies aimed at defining the quantitative and qualitative characteristics, as well as the timing and methods of dietary intakes (food windows), has the potential to prevent or delay the onset of obesity. However, further, longer-term, and more in-depth studies are still required for their precise definition. 

#### 2.1.2. Therapeutic Strategies for Gut Microbiota Dysbiosis

A microbial intestinal imbalance has been associated with diabesity [15]. The gut microbiota participates in the metabolism, influencing the energy balance, glucose metabolism, and low-grade inflammation associated with obesity [107]. The ancestral human diet was essentially made of vegetables, consisting of complex carbohydrates fermented by the intestinal microbiota to produce energy [108]. Western diets are generally low in fiber and rich in fat and digestible sugars [109], as well as a high uptake of SFA [110], which can lead to an alteration in the composition of the gut microbiota and obesity and diabetes [110]. Intestinal dysbiosis alters the production of gastrointestinal factors related to satiety and metabolism, with a consequent increase in fat storage promoting the development of obesity, T2D, and MS [111,112]. Efforts are intensifying to design or replenish the microbiota to prevent or cure dysbiosis-related complications. The composition of the gut microbiota can be modified by several means, including the use of live bacteria (probiotics), specific nutrients acting as substrates for bacterial growth (prebiotics), antibiotics, or fecal microbiota transplantation (FMT) [113]. Bariatric surgeries (gastric bypass) cause rapid adaptation of the microbiota [114], and the gut microbiota shows resilience after these procedures [115]. Regarding FMT, its use is still controversial [116], and it should be noted that its long-term effectiveness remains elusive when performed repeatedly [117]. A strategic approach to modulating the microbiota could involve the rational design of personalized diets [118]. This approach considers both the rapid and reproducible reactivity of the microbiota to dietary intervention [119] and, in the case of metabolic diseases, the possibility of knowing the composition of the microbiota and predicting individual responses to dietary intervention [120].

#### 2.1.3. Gut Microbiota Modulation in Obesity by Pre-Probiotics

The control of the intestinal microbiota using prebiotics, probiotics, or diet plays a key role in obesity therapies [121,122,123].

Prebiotics are defined as a “nonviable food component that is selectively utilized by microorganisms, conferring a health benefit on the host associated with the modulation of the microbiota” [124,125]. Several sugar prebiotics (oligofructose, inulin, fructooligosaccharides, galactooligosaccharides, and resistant starch) have demonstrated therapeutic effects on obesity [126,127,128]. Different peptides secreted by enteroendocrine cells of the gastrointestinal tract, such as glucagon-like peptide-1 (GLP-1), peptide YY (PYY), gastric inhibitory peptide (GIP), and ghrelin, are involved in the regulation of energy homeostasis and capable of modulating food intake and energy expenditure linked to obesity [129,130]. In addition, an interaction was observed between the fermentative processes of non-digestible carbohydrates (inulin-type fructans and resistant starches) by the intestinal microbiota and the improvement of metabolic disorders [131]. The positive effects of prebiotics concern serum lipids, inflammatory markers (IL-6), glucose homeostasis, and blood pressure [128,132].

The prebiotic effect, understood as selective stimulation of the development and/or activity of one or a limited number of microbial species [133], has been extended to the activity of polyphenols [125]. The genome of intestinal microbes encodes different enzymes that contribute to the bioavailability and bioactivity of unabsorbed polyphenols [134]. Polyphenols exert potential prebiotic effects by selectively stimulating beneficial bacteria and reducing the incidence of metabolic disorders [135,136] and cardiometabolic risks [137]. Some polyphenols may inhibit the growth of harmful bacteria, such as *Helicobacter pylori*, *Staphylococcus aureus*, *Escherichia coli*, *Salmonella typhimurium*, *Listeria monocytogenes*, and *Pseudomonas aeruginosa*, as well as the hepatitis C virus and *Candida* [138]. Other polyphenols, by contrast, may change the composition of the microbiome in favor of beneficial bacteria, including *Bifidobacterium* spp., *Lactobacillus* spp., *Akkermansia muciniphila*, and *Faecal bacterium prausnitzii*, and improve the ratio of *Firmicutes to Bacteroidetes* [138,139]. 

Alterations in the gut microbiota influence gene expression involved in metabolic and inflammatory homeostasis [140]. In MS induced by high-fat diets, an important role is attributed to the variation of the intestinal microbiota and the consequent inflammation [141]. In particular, a study of 14 diets in mice with different fat, protein, and fiber contents highlighted the prevalent action of a lack of soluble fiber (inulin) in promoting obesity. This suggests that inulin prevents inflammation by supporting intestinal homeostasis mediated by the microbiota [142]. In pigs, feeding inulin has been shown to limit the adverse effects of a high-fat diet by diversifying the microbial population, increasing the oxidation of fatty acids, and suppressing their synthesis [143].

The change in the composition of the intestinal microbiota by prebiotics (inulin type-fructan and oligofructose) leads to a significant reduction in food intake, body weight gain, and the development of fat mass. This is associated with greater production and secretion of anorexigenic peptides (GLP-1 and PYY) and a reduction of the orexigenic peptide (ghrelin) [144,145]. Dietary treatment with inulin-type fructans in obese women (16 g/day for 3 months) resulted in an increase in the number of *F. prausnitzii* in the feces [146]. The treatment with inulin or oligofructose (15 g/day for 15 days) increased the proportion of *Bifidobacterium*; conversely, the proportion of *Bacteroides*, *Clostridia*, and *Fusobacteria* decreased in response to oligofructose, and the population of gram-positive cocci decreased in response to inulin [147]. There is numerous evidence that the alteration of the proportion of *Bacteroidetes* and *Firmicutes* leads to the development of obesity [123]. It has been found that the relationship between *Firmicutes* and *Bacteroidetes* changes in favor of *Bacteroidetes* in overweight and obese subjects [148].

The individual enterotype seems to influence weight loss in relation to diet [149]. In diets rich in fiber and whole grains, the high ratio of *Prevotella/Bacterioides* conditions a greater susceptibility to weight loss [150]. 

Probiotics are described as “live microbial food supplements that have a beneficial effect on the host animal by improving the intestinal microbial balance” [151]. According to FAO/WHO (2001) [152], “probiotics are mono or mixed cultures of living organisms, which, when administered in adequate amounts, confer a health benefit to the host”. In animals, probiotics are gaining importance as potential alternatives to antibiotics to improve productive efficiency [153]. In humans, probiotics play a fundamental role in epithelial integrity and, consequently, have a beneficial effect on reducing gastrointestinal diseases [154]. In particular, *Lactobacillus* spp. and *Bifidobacterium* spp. have beneficial effects on obesity by improving the microbiota, reducing plasmatic lipids and pro-inflammatory genes, and increasing the production of short-chain fatty acids (SCFAs) [145,155,156,157].

In a human study, probiotic supplementation was able to prevent high fat content and overfeeding-induced insulin resistance compared to the control group [158]. In addition, the integration of probiotics appears to improve hypertension through the amelioration of lipid profiles and regulation of insulin sensitivity [159].

Several studies have reported superior benefits with the use of a mix of probiotic strains and combinations of pre-probiotics, compared to single use [156,160,161].

#### 2.1.4. Gut Microbiota Modulation in Obesity by Dietary Fatty Acids

Diet is one of the major factors contributing to obesity, making it a potential target for treating gut microbiota dysbiosis. In the treatment of overweight patients with low-calorie diets, the gut microbiota has been considered a prognostic factor for weight loss and improvements in metabolism and inflammatory profile [121]. High-fat diets have negative effects on microbiota modulation [162]. Fatty acids may influence energy production, alter nutrient absorption, and produce toxic compounds for cells, leading to the inhibition of growth and bacterial death. Dietary fat and fatty acid composition can reduce the number of species and genera of bacteria related to overweight [119,163]. The high consumption of saturated fatty acids (SFA) can induce a profile of overweight-related microbiota through the decrease of *Bacteroides*, *Prevotella*, *Lactobacillus* ssp., and *Bifidobacterium* spp. [164,165,166].

A prebiotic effect can be extended to the activity of fatty acids [125,167]. The quality of fatty acids in the diet may affect the composition of the intestinal gut microbiota and, consequently, the host metabolism [168]. The presence of double bonds increases the effectiveness of the unsaturated fatty acids (FAs) [169]. The dietary composition of FAs influences inflammation by altering the availability of substrates for pro-inflammatory eicosanoid (C20:4 n-6 arachidonic acid) or anti-inflammatory agents (eicosapentaenoic acid, EPA—C20:5n-3-, and docosahexaenoic acid, DHA-C2:6 n-3) [170].

Food polyunsaturated fatty acids (PUFA) n-3 have been shown to protect rats from dysbiosis by reversing bacterial proliferation resulting from the intake of n-6 PUFA [171]. In contrast, the consumption of PUFA n-6 results in the depletion of *Bacteroidetes* and *Firmicutes phyla*, an increase in BMI, and the infiltration of inflammatory cells in the ileum. High bacterial growth in the small intestine can be a source of abdominal pain, swelling, and poor absorption of fat, protein, and vitamins (such as B12), accompanied by dysbiosis [172].

The high consumption of n-6 PUFA causes an imbalance in the ratio n-3/n-6, leading to an increase in the concentrations of arachidonic acid and consequent chronic inflammation associated with obesity [173]. The ratio n-6/n-3 in the Western diet is close to 20:1, instead of the recommended 1:1. In addition, a high intake of n-6 PUFA during the prenatal period is associated with increased adiposity in offspring [174]. 

Animal studies have revealed that the consumption of n-3 PUFA and conjugated linoleic acid (CLA) is beneficial for the microbiota, unlike n-6 PUFA and SFA. Food supplementation with CLA improves gut microbiota by increasing the bacterial population and can contribute to the control of obesity [163]. Dietary supplementation with PUFA n-3 can also improve the composition of the intestinal microbiota [175] by increasing the production of the intestinal alkaline phosphatase enzyme [176]. This decreases intestinal production and permeability of LPS, reducing metabolic endotoxemia and inflammation [177]. 

Since PUFA n-3 cannot be synthesized by the organism, they must be obtained from food sources [178,179] of plant origin, in the form of α-linolenic acids (ALA), or from certain species of fish, in the form of EPA and DHA [179,180,181]. The primary sources of EPA and DHA are seafood, such as sardines, salmon, tuna, mackerel, and herring [179,182]. The increase in circulating PUFA n-3 through dietary supplementation can reduce the incidence of MS induced by obesity, including insulin resistance, hypertension, and dyslipidemia [183].

High concentrations of PUFA n-3 have been associated with improved insulin sensitivity [184,185]. It has been shown that taking PUFA n-3 reduces systemic inflammatory markers and the circulation of lipids in the blood and lowers the risk of T2D [186]. Intake of n-3 fatty acids reduces circulating inflammatory cytokines such as TNF-α, IL-1, and IL-6 [187]. EPA and DHA regulate the production of anti-inflammatory eicosanoids (PG 3 series, LT, and resolvins) and gene expression of cytokines, such as the transcription factor PPAR-γ. The activation of PPAR γ can directly generate the production of anti-inflammatory cytokines and suppress the activation of the pro-inflammatory transcription factor NF-κB. The n-3 fatty acids can modulate the synthesis of triacylglycerols through the reduction of the availability of fatty acids (lower lipogenesis de novo), the reduction of the activity of enzymes that synthesize triglycerides (TG), or by increasing the synthesis of phospholipids [188,189]. 

The role of PUFA n-3, particularly EPA and DHA, in the treatment of obesity has been studied, but the results are contradictory [190,191]. A meta-analysis study suggested that the consumption of fish or encapsulated fish oil (rich in PUFA n-3) is related to slight reductions in body weight and waist circumference [192]. However, another study found that fish oil has no effect on the reduction of body weight and BMI in overweight or obese individuals [193]. A recent meta-analysis has also shown that n-3 PUFA has no significant incidence on weight loss, while it could effectively reduce waist circumference and triacylglycerol (TAG) levels in overweight and obese adults [194]. These conflicting results suggest the need for a more in-depth understanding of the possible mechanisms of action of n-3 PUFA on weight reduction [192] and a large-scale investigation over a long period to draw definitive conclusions [193]. Polyphenols from the Mediterranean diet are known to exert modulatory effects on the gut microbiota and gut-liver axis [21].

## 3. Beneficial Effects of Antioxidants in Metabolic Syndrome

Organisms in biological systems create antioxidant defense mechanisms to counteract oxidative stress—enzymatic (catalase, superoxide dismutase, glutathione peroxidase) and non-enzymatic (glutathione, selenium, vitamins: A, β-carotene; C, ascorbic acid; E, α-tocopherol)—that regulate the levels of free radicals/oxidants to maintain redox physiological homeostasis [195].

Antioxidants of food origin (vitamins A, C, E, minerals) are defined as a secondary defense system [196,197]. Carotenoids (beta-carotene, lycopene, lutein, and zeaxanthin) are regarded as one of the most efficient singlet quenchers of oxygen, as well as ROS scavengers operating in cellular lipid bilayers, and have been reported to be useful in the prevention of metabolic diseases, cardiovascular disease, and cancer [198,199]. 

Selenium (Se), in the form of selenoproteins, is an essential micronutrient that plays an important role in health. Low levels of Se are associated with an increased risk of metabolic disorders, mainly linked to limited antioxidant defense resulting from Se deficiency [200]. Tocopherols (α-tocopherol, ɣ-tocopherol, δ-tocopherol, and ɣ-tocotrienol) protect membrane lipids and show both antioxidant and anti-inflammatory activity [201]. Endogenous antioxidant defense mechanisms act together with exogenous antioxidants, such as food polyphenols and vitamins [202].

Among the bioactive antioxidants of food origin, mainly present in fruits and vegetables, medicinal plants, and plant-based foods, an important role is attributed to the polyphenols, formed by flavonoids such as flavones, flavonols, flavanols, flavanones, isoflavones, anthocyanins, and non-flavonoids such as phenolic acids, stilbene derivatives, and lignans [179]. The antioxidant activity of polyphenols is linked to the number and position of the -OH groups on the aromatic ring and, thus, to the replacement of hydroxyl groups in the aromatic ring [203].

The preferred management of MS involves restoring the body’s redox balance, the consumption of a diet low in fats, low in carbohydrates, and salt, reducing alcohol intake, and increasing the intake of fruit, vegetables, and antioxidants [66]. Abnormal systemic oxidative stress, characterized by an increase in free radical production and redox imbalance, is a key feature of MS. Oxidative stress is implicated in various diseases, including diabesity, cardiovascular and neurological disorders, and the aging process [12,204]. Antioxidants play a crucial role in controlling oxidation by blocking the formation of free radicals or interrupting the propagation of the free radical chain reaction. Antioxidant mechanisms include the elimination of the species that start the peroxidation, chelating metal ions, prevention of peroxide formation by turning off •O_2_, interruption of chain reaction in autoxidation, reduction of oxygen concentrations, and enhancement of antioxidant enzyme activities [8]. Effective antioxidants can break the chain reaction of free radicals, as they contain one or more aromatic rings (often phenolic) with one or more -OH groups. These compounds can provide H• to free radicals during oxidation [205,206]. Phenolic acids act as antioxidants by trapping free radicals, while flavonoids can scavenge free radicals and chelate metals [205]. 

### 3.1. The Potential Role of Polyphenols in Metabolic Disorders

**Figure 1:**
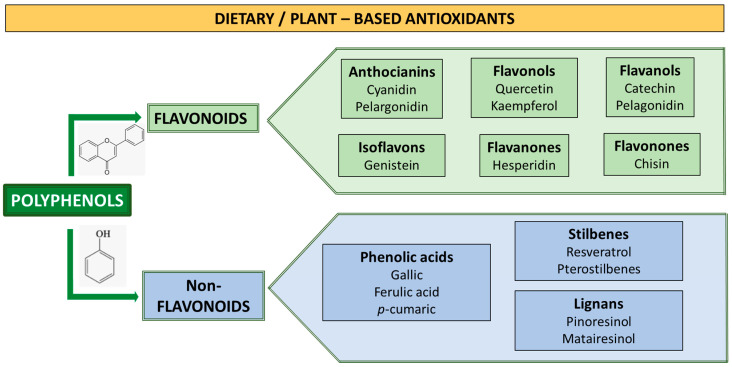
Main categories of natural phenolic compounds.

In order to prevent the development of or clinically intervene in MS disorders, emerging therapeutic strategies involve the exploration of natural plant-based antioxidants. The beneficial health effects are attributed to various dietary elements, including the secondary metabolites of plants. Polyphenolic compounds represent one of the largest groups of bioactive compounds derived from plants and are associated with biological actions involved in the prevention and control of chronic diseases [207,208,209]. Polyphenols are the most abundant antioxidants in the human diet (approximately 1 g/d) [210,211]. Polyphenols are the largest group of phytochemicals containing phenol rings and ligand groups, mainly hydroxyl linked with carbon sites. Over ten thousand natural polyphenols with diverse properties and bioavailability have been identified [210,212,213] and classified into flavonoids and nonflavonoids (Figure 1) based on their structural characteristics [210]. Flavonoids have a skeletal structure of 15-carbon atoms with two aromatic rings connected by a different pyronic ring C, organized in a C6-C3-C6 configuration. Flavonoids include several subgroups: flavonols, flavones, isoflavones, flavanons, anthocyanidines, flavan-3-oils, and minor subclasses such as chalcones chalk [210]. Non-flavonoids include phenolic acids, hydroxyhymamic acids, stilbenes, lignans, and coumarins [214]. Two-thirds of food polyphenols are flavonoids, while one-third come from phenolic acids [215]. Flavonoids are part of the animal and human diet; they are not synthesized within the body and are provided by plants [216]. The primary sources of flavonoids include fruit, vegetables, dark chocolate, olive oil, tea, coffee, and red wine [210,217]. In foods, the most common class of flavonoids is flavonols, which are present as glycosides in fruits and as derivatives of quercetin in vegetables [218].

Polyphenols have brought attention to the low bioavailability/high bioactivity paradox [219,220]. Polyphenols are subjected to gastrointestinal digestion, resulting in important changes in content and antioxidant activity [221]. Dietary metabolites of polyphenols, including resveratrol, curcumin, quercetin, rutin, genistein, daidzein, ellagitannins, and proanthocyanidins, produced through phase I and phase II metabolic reactions and gut microbiota-mediated biotransformation, reveal significant activity relative to the progenitor molecules in terms of cell signaling and pharmacokinetic activity [23,219,222,223]. Less than 5% of the total polyphenolic intake is absorbed and reaches plasma unchanged [224]; the majority is metabolized by enterocytes and by phase I and phase II enzymatic reactions [223,225,226,227,228]. Furthermore, polyphenols undergo a significant transformation by the intestinal microbiota, forming novel chemical structures with higher bioactivity than the parent [229,230], and can easily enter the systemic bloodstream [222,231,232]. 

Many studies have emphasized the potential role of polyphenols and their metabolites in the prevention of various diseases, including obesity, diabetes, cardiovascular diseases, and neurodegenerative diseases [21,22,23,24]. 

#### 3.1.1. Dietary Polyphenols and Diabesity

Several studies indicate that polyphenols can delay or prevent the onset of MS by decreasing body weight, blood pressure, and glycemia and improving lipid metabolism [228,233]. These compounds may modulate either carbohydrate digestion or glucose metabolism [234,235,236].

The intake of polyphenols, particularly flavonoids, appears to be an effective intervention in the prevention of T2D. They can inhibit the activity of carbohydrate digestive enzymes, such as α-amylase and α-glucosidase [237,238], by lowering the starch digestive rate and affecting the bioavailability of carbohydrates and subsequent postprandial glucose levels [236]. Phenolic compounds can inhibit the transport of glucose into the intestinal cells at the lumen level, involving glucose transporter 2 (GLUT2) and glucose transporter coupled with sodium 1 (SGLT1) in enterocytes, and stimulate glucose clearance in the blood by transporting glucose 4 (GLUT4) into muscle cells [239,240]. Polyphenolic metabolites can also counteract oxidative stress in pancreatic β cells by strengthening insulin secretion [241,242]. Polyphenols promote glucose uptake by improving insulin sensitivity through the activation of AMP-activated protein kinase pathways [243]. In the liver, foods rich in polyphenols prevent gluconeogenesis and stimulate glycogenesis [243], improving insulin-reduction pathways indirectly by reducing glucose synthesis [244]. 

The intake of flavanols has shown beneficial effects on the improvement of insulin resistance, systemic inflammation, oxidative stress, and other cardiometabolic risk factors [245,246,247]. The intake of flavonones, dihydroflavonols, and stilbenes was associated with a reduced risk of diabetes in elderly subjects [248]. 

Among flavonols, primary food sources include cocoa, chocolate, green tea, and red wine [210]. In a meta-analysis study evaluating the effects of cocoa intake for 2–18 weeks in 1106 individuals, including people with diabetes, overweight, and hypertension, the homeostatic model of insulin resistance decreased by 0.94 points [245]. An acute short-term intake (18 weeks) of cocoa, chocolate, and flavan-3-ols also reduced insulin resistance [246]. 

Catechins are considered the most important flavones. Among these, epigallocatechin-3-gallate (EGCG) is the stronger and more abundant catechin (65% of the total content) in green tea [249]. A clinical study has shown that administration of green tea for 12 weeks containing 528.8 mg of catechins reduced insulin levels in T2D patients, although no significant difference in fasting glucose levels or glycosylated hemoglobin was observed [250]. Another clinical study found that supplementation with 1500 mg of green tea extract [856 mg of epigallocatechin gallate (EGCG)] for 16 weeks caused a significant reduction in fasting insulin, insulin resistance, and glycated hemoglobin in obese individuals with T2D [251]. 

Several studies have revealed a reverse association between catechin intake and obesity [252,253]. A clinical study has shown that consumption of Oolong tea (containing 690 mg vs. 22 mg of catechins) for 12 weeks can cause a significant reduction in body weight, BMI, and adipose tissue [254]. Another study found that consumption of catechin-rich drinks for three months can cause a significant reduction in body fat percentage, body weight, intra-abdominal fat, and waist circumference [255].

However, some studies did not observe any improvement in anthropometric weight after catechin supplementation [256,257], probably due to the confusing effect of caffeine in green tea, which has been shown to increase energy expenditure and thermogenesis, thus affecting body weight [258]. Other results show no positive effects of polyphenols on weight control. Intake of apple juice (750 mL/day with 802 mg polyphenols) for 4 weeks does not affect body weight, body mass index, or girth of life in obese individuals [259], as does the consumption of orange juice (500 mL/day with 250 mg anthocyanin) for 12 weeks, which does not induce any weight loss [260]. Again, resveratrol, mainly contained in grapes, wine, and some red fruit, has been proposed as a regulator of weight as it can inhibit the proliferation of adipocytes and lipogenesis, stimulate lipolysis, and beta-oxidation of fatty acids [252]. However, it has provided consistent results of weight reduction only if consumed as a food integrator [261,262]. The conflicting results are mainly due to the wide variability of the studies.

In addition, it should be underlined that studies on the prevention and treatment of MS in humans are limited. Clinical studies have also found that the use of purified individual nutritional molecules has no positive results, such as in the inversion of obesity or in diseases related to obesity [263,264]. Therefore, treatment with multiple combinations of natural products can involve a synergistic activity that can increase their bioavailability and act on multiple molecular targets, offering advantages over pure chemical products. Better results in metabolic control can be obtained with a diet containing a different subclass of polyphenols, which can act synergistically compared to a single food or phenolic compound; this could be achieved by adhering to the Mediterranean diet. 

#### 3.1.2. Role of Mediterranean Diet and Ingredients in Metabolic Syndrome

**Figure 2:**
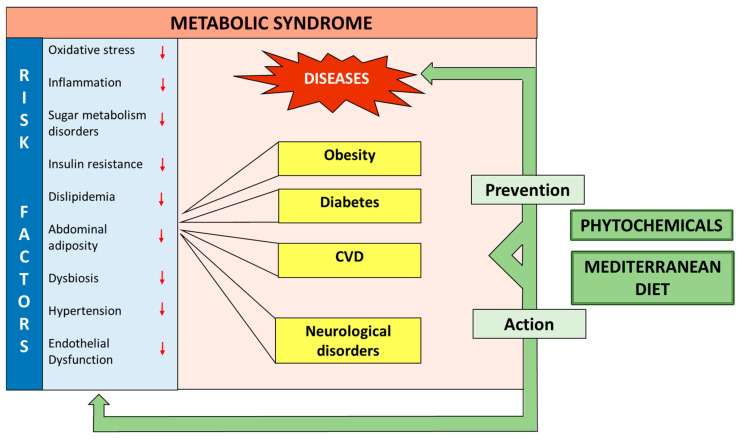
Beneficial effects of Mediterranean diet and phytochemicals in the prevention of metabolic syndrome and associated diseases.

In the prevention of chronic diseases, including obesity, diabetes, cardiovascular, and neurological diseases, there has been a growing interest in the beneficial effects of plant-based diets [265,266,267] and the Mediterranean diet (MedD) (Figure 2), to which many health benefits are associated [268]. Randomized trials have shown that MedD is able to reverse the condition of metabolic syndrome and may be useful in reducing the risks of central obesity and hyperglycemia in people at high risk of cardiovascular disease [21,269]. 

The high consumption of MedD plant foods is associated with beneficial metabolomic profiles related to the microbiome [270]. Specific changes in the gut microbiota have been associated not only with obesity but also with type 2 diabetes. Several polyphenols may change the composition of the microbiome in favor of beneficial bacteria, including *Bifidobacterium* spp., *Lactobacillus* spp., *Akkermansia muciniphila*, and *Faecal bacterium prausnitzii*, and improve the ratio of *Firmicutes* to *Bacteroidetes* [138,271]. The biological effects of polyphenols are linked to their bioavailability within the human body, which is strongly influenced by the gut microbiota and its ability to transform food polyphenols into a wide range of different metabolites [272]. These metabolites can be absorbed more efficiently by intestinal epithelial cells and may have greater bioactivity than their parent molecules, providing beneficial effects for the host [229].

The MedD is characterized by a high intake of fruits, vegetables, cereals, legumes, nuts, and olive oil; a moderate consumption of fish, dairy products, and wine; and low amounts of red and processed meat, butter, cream, and sugary drinks. Foods such as olive oil, legumes, fruit, vegetables, red wine, and nuts are rich in polyphenols [268,273]. The mechanisms of action of MedD on metabolic processes are poorly defined, but the inflammatory system seems to be involved. It has been observed that the diet can determine a reduction of blood lipids, inflammatory and oxidative stress markers, improvement of insulin sensitivity, enhancement of endothelial function, and antithrombotic function [274,275]. Furthermore, the beneficial effects are linked to the characteristics of the type of food consumed [276]. 

Lipid sources in the MedD include foods rich in unsaturated fatty acids and antioxidants, such as olive oil, fish, and nuts [277,278] MedD is recognized for its antioxidant and anti-inflammatory actions [279,280] attributed to the high constituents of phenolic compounds, mono- and poly-unsaturated fatty acids, and fiber [275,279]. It is known for its protective effect against insulin resistance [278] and its role in glycemic and T2D control [281]. A study on PREDIMED highlighted an inverse relationship between the total dietary intake of polyphenols and bioactive constituents derived from different sources as components of the MedD and the risk of mortality [248].

#### Extra-Virgin Olive Oil

Olive oil (virgin and extra virgin) is considered the most important constituent of MedD. It contains monounsaturated fatty acids (MUFAs) and polyphenols, including secondary plant metabolites such as oleuropein, tyrosol, hydroxytyrosol, secoirodoids, and lignans [277]. The polyphenol content in olive oil varies between 40 and 1000 ppm, influenced by factors such as olive cultivar, ripening time, climate, and extraction process [282]. Secoiridoids are important phenols in olive oil [283]. They are metabolized by hydrolysis in the gastrointestinal tract, producing tyrosol and hydroxytyrosol from ligstroside and oleuropein, respectively. Tyrosol has been implicated in antioxidant defenses, leading to increased phosphorylation of protein kinase B (AKT), endothelial nitric oxide synthase (eNOS), and sirtuin1 against ischemic stress [284]. 

In patients with MS, a MedD enriched with extra virgin olive oil enhances plasmatic antioxidant capabilities, leading to increased levels and activity of superoxide dismutase and catalase, elevated nitrate levels, and decreased activity of xanthine oxidase [285]. Olive oil has the potential to modulate insulin signaling. Oleic acid, a component of olive oil, improves the fluidity of the cell membrane and insulin receptors [286]. It can also mitigate the hyperactivity of beta cells and insulin resistance in individuals with hypertriglyceridemia [277]. The supplementation of oleuropein and hydroxytyrosol further enhances insulin secretion and insulin sensitivity [287].

In overweight patients with T2D, daily intake of polyphenol-rich olive oil for 8 weeks significantly decreased fasting blood glucose and glycated hemoglobin (Hba1c) [288]. MedD supplemented with extra virgin olive oil (EVOO) has beneficial effects on glucose metabolism [281]. It reduces fasting blood glucose, improves insulin resistance, lowers inflammatory biomarkers [289], and reduces the risk of T2D by 40% [281]. Olive oil polyphenols act through various mechanisms to affect glucose metabolism, including the inhibition of carbohydrate digestion and absorption, reduction of glucose release from the liver, stimulation of glucose processes in peripheral tissues, production of advanced glycosylated end products [226,243], and prevention of abnormal postprandial lipemia [290].

Clinical studies have shown that virgin olive oil can also restore intestinal microbiome dysbiosis in obese patients [291] and modulate their gut microbiota, leading to improved insulin sensitivity [292].

Overall, a MedD integrated with EVOO, through the content of polyphenols, can have an effect on lowering central obesity [269] and on the maintenance or loss of body weight [293].

#### Fish/Seafood

Fish and seafood in the MedD include sardines, mackerel, mussels, octopus, oysters, salmon, sea bass, shrimp, squid, and tuna. PUFA n-3, specifically eicosapentaenoic acid and docosahexaenoic acid, are considered the most important bioactive components of fish that can affect health [294,295]. Fish and other seafood are complete protein sources that can reduce glycemic response [296]. The amino acid composition of fish proteins can increase glucose uptake by the muscles through improved insulin sensitivity [297]. Some studies report a beneficial effect of fish intake on the glycemic state [298,299], while others do not [300].

Dietary recommendations suggest eating at least two servings of fish a week, with one of them being fatty fish [301]. A European study in adult men and women found that total intake of both white and fatty fish was associated with a 25% reduction in the risk of diabetes (odds ratio: 0.75) [302]. A meta-analysis study revealed that the consumption of fatty fish reduces the risk of T2D, while no significant association has been found for lean fish [303]. Other studies confirm a protective effect of fatty fish consumption on the development of T2D [304,305].

#### Fruits and Vegetables

A diet rich in fruits and vegetables is associated with a reduced risk of diabetes and obesity [306,307]. Fruit and vegetables are good sources of fiber, which stimulates the proper digestion of food. Soluble fibers, through the formation of a denser chyme, delay gastric emptying, maintain a high level of satiety, and reduce the amount of food ingested. The impact of fruit and vegetable fiber on gastric emptying and satiety directly influences leptin, neuropeptide Y, gastric inhibitory peptide, and neurotrophic factor derived from the brain [308]. As a result, it hinders weight gain and obesity. The formation of a denser gelatinous chyme by the soluble fiber leads to a reduction in the absorption of glucose, which, in turn, determines a lower postprandial peak of glucose and insulin peaks [309]. This mechanism is improved with the consumption of fruits with a low glycemic index (apples, oranges, pears, and berries) because it greatly reduces insulin resistance and post-prandial concentrations of glucose in the blood [310]. 

Insoluble fiber reaches the colic tract of the gastrointestinal system, serving as a substrate for strains of intestinal microbiota (mainly *bifidobacteria*). These strains degrade long, indigested polysaccharide chains and release SCFAs in the bloodstream. SCFAs in the blood circulation affect the preservation of glucose in muscles, liver, and fats, regulate the immune system, and can reduce the risk of diabetes [311,312]. Acetate reaches the brain and decreases appetite and food consumption. Propionic and butyric acid can inhibit the activity of β-hydroxy β-methylglutaryl-Coa reductase and cholesterol synthesis [313], modulating liver glucose metabolism, adipogenesis, and leptin production [314]. Fruits such as apples, pears, apricots, cherries, berries, and grapes, as well as vegetables including carrot, tomato, onion, garlic, cabbage, and celery, contain high amounts of polyphenols (up to 200–300 mg per 100 g) [315]. These polyphenols may reduce the risk of T2D. For example, anthocyanins (found in red and violet fruits), flavan-3-oils (present in berries, grapes, etc.), and flavanones (found in citrus fruits) have shown robust inhibitory activity against α-glucosidase and α-amylase at the intestinal level. This leads to a delay/inhibition of carbohydrate digestion and plays a key role in improving insulin sensitivity [243,316]. All these effects lead to a reduction in the risk factors associated with insulin resistance, T2D, increased body weight, and obesity. 

#### Grains

The MedD derives approximately 50% of its daily calories from carbohydrates, with the majority of these carbohydrates coming from legumes, non-refined grains, and fruit. These sources have a lower glycemic index compared to added sugars or refined grains, leading to a slower release of glucose during digestion. 

Grains are included in products such as bread, pasta, crackers, and cereals. The consumption of whole grains has a protective effect against T2D [317], is associated with low concentrations of fasting glucose and insulin [318,319], and may help reduce the risk of heart disease and certain cancers [320].

Observational studies and randomized controlled studies have shown that a higher intake of whole grains is linked to a lower risk of obesity and weight gain [321,322]. An inverse relationship has been found between whole grain intake and BMI, or abdominal obesity [322,323]. The possible mechanisms involved can include appetite suppression and weight loss, reduced glycemic load in the diet, improved insulin sensitivity, and modulation of the intestinal microbiota [321,324]. Certain types of dietary fiber may affect weight status. For example, beta-glucans and type 4 resistant starch have been shown to increase satiety [325,326], thus reducing energy intake and mitigating postprandial glycemic responses [327].

Fermentable fibers are processed by bacteria in the colon, producing SCFAs [328], which also influence body weight and composition through liver and peripheral glucose and lipid oxidation, stimulating peptide secretion of the intestinal hormones PYY and GLP-1 [329]. Additionally, SCFAs can alter the composition of the gut microbiota, which, in turn, can influence obesity [330]. The bioactive components of whole grains, such as lignans and phytosterols, have been shown to exert metabolic effects that can affect body weight and adiposity [331,332,333]. Compared to refined grains, whole grains are richer in magnesium and antioxidants, which have been associated with lower levels of glucose and insulin in fasting and improved insulin sensitivity [334,335].

#### Legumes

Legumes, such as chickpeas, lentils, beans, and peas, are commonly found in MedD. They are rich in protein (ranging from 20% in beans and peas up to 38–40% in soybeans), soluble dietary fiber fractions, phytosterols, flavones, and minerals [336,337]. Legumes have a low glycemic index (around 50) and contain bioactive compounds, such as genistein and daidzein, alpha-amylase inhibitors, and alpha-glucosidase inhibitors, which show antioxidant activity along with anti-inflammatory properties [336]. A meta-analysis study showed that increasing fiber consumption to about 17 g/day reduces systolic pressure (SBP) by 1.15 mmHg and diastolic pressure (DBP) by 1.65 mmHg [338,339]. This suggests a probable linear relationship between legume consumption and fasting blood TG, HDL-C, liver enzymes, glucose, insulin, and C-peptide. Randomized controlled studies have shown that a legume-rich diet has beneficial effects on insulin resistance and inflammation biomarkers, cholesterol levels, body weight, and central obesity [166,339,340]. 

#### Nuts

Nuts are also components of MedD. They contain MUFAs and PUFAs, as well as vitamin E, vitamin B2, folate, and fiber. Many seeds and nuts are rich sources of polyphenols; ellagitannins are contained in chestnuts and walnuts, whereas proanthocyanidins are more present in hazelnuts, pecans, and almonds. Flax seeds have more lignans [273]. Indirect evidence indicates that ellagic acid, which is present in large quantities in several nuts (mostly walnuts), has beneficial effects on glucose metabolism and diabetes control [341]. Nut consumption seems to exert a protective effect on cardiometabolic diseases, improving concentrations of fasting glucose, total cholesterol, and LDL-C [342]. Nut intake has been inversely associated with inflammatory markers and glucose/insulin homeostasis, mediated by some adiposity indexes (BMI and waist circumference) [343]. Clinical studies also indicate that the effect of nut consumption on adiposity is still unresolved and requires further investigation [344].

#### Wine

The MedD is characterized by a moderate consumption of wine (1–2 drinks/day or ~150–300 mL/day) [345] during principal meals) [346,347]. Red wine is known to contain 10 times more phenolic compounds than white wine [348] and includes flavonols (quercetin and myricetin), flavanols (catechin and epicatechin), anthocyanin, and stilbenes (resveratrol).

Resveratrol, highly abundant in red wine, exhibits a superior capacity for eliminating radicals compared to vitamin C, vitamin E, and propyl gallate [349]. It plays an important role in reducing obesity [350] and improving glycemic control in individuals with insulin resistance or diabetes [351,352], including obese men [351] and those with MS [353].

Randomized clinical studies have shown that red wine enhances glucose metabolism and significantly reduces insulin resistance in T2D patients, as assessed by the homeostatic model of insulin resistance (HOMA-IR) [354,355]. However, other studies do not entirely confirm these observations [356,357], suggesting the need for further trials to better understand the antidiabetic properties of red wine poliphenols.

#### 3.1.3. Cardiometabolic Protection by Polyphenols

MS has a significant impact on public health, as it has been associated with an increasing risk of cardiovascular diseases, affecting millions of people in the modern world and constituting the leading cause of death in Western countries [358]. Oxidative stress, characterized by an imbalance between the generation of ROS and RNS and antioxidant defense systems, is the mechanism that leads to molecular and tissue damage, contributing to the development of CVDs.

Several experimental and clinical studies have revealed that risk factors for cardiovascular diseases, including genetic predispositions, high levels of cholesterol, hypertension, diabetes, and obesity, are associated with an increase in oxidative stress [359,360,361]. Additionally, the heart is characterized by low concentrations of antioxidants, making it more susceptible to damage, particularly in macromolecules such as DNA, proteins, and cell lipids [362].

Dyslipidemia, inflammation, and atherosclerosis are the main causes of CVD [363,364]. Inflammation is consistently present in the processes of atherogenesis and thrombosis, increasing the risk of infarction and strokes. This is driven by the interaction between adhesion molecules, cytokines, circulating mononuclear cells, low-density lipoprotein cholesterol (LDL-C), and the vascular endothelium. The inflammatory process affects the formation of atherosclerotic plaques, leading to an increase in vessel thickness, a decrease in the lumen size, and altered blood flow [365]. The rupture of the plaque results in the formation of an embolus [363,366]. Both the plaque and embolus can impact blood flow in small vessels, causing ischemia in organs [363] and subsequent complications such as coronary heart disease (CHD), stroke, and peripheral arterial disease [366].

CHD involves ischemia in the coronary artery, either due to the accumulation of atherosclerotic plaques or due to the migration of emboli into the coronary artery. This can lead to a reduction in blood flow, stiffening of blood vessels, and a consequent decrease in cardiac output or cell death [367]. Peripheral arterial disease is generally associated with the formation of atheroma plaques in peripheral arteries or an embolic process, resulting in limb ischemia, pain, and reduced movement [368].

Among the biomarkers of CVD, C-reactive protein (CRP) serves as an acute inflammatory biomarker and is used in predicting the risk of atherosclerosis. It is a strong predictor of CVD risk [369]. Lipoproteins such as LDL, VLDL, and HDL are also crucial predictors of CVD [370]. A high level of HDL is considered a protective factor against CVD [371]. 

In recent years, the use of polyphenols has become common in the treatment and prevention of CVD [372]. The cardioprotective properties of polyphenols are associated with the reduction of blood pressure [373], the improvement of endothelial tissue function [374], antiplatelet activity by inhibiting platelet aggregation [375], reduction of LDL, and reduction of the inflammatory response [376].

Different cardiovascular diseases can be linked to gut microbiota dysbiosis, which affects dyslipidemia, inflammation, and atherosclerosis [377,378]. It has been reported that dysbiosis exerts pro-atherosclerotic effects by altering the generation of various metabolites [379]. Trimethylamine-N-oxide, an intestinal metabolite, promotes the development of cardiovascular disease by affecting metabolism [380]. Intestinal dysbiosis contributes to hypertension through vasoconstriction induced by oxidized LDL (ox-LDL) and promotes the expression of pro-inflammatory cytokines and the formation of foamy cells [381]. The restoration of the composition and function of the intestinal microbiota can have a significant impact on the improvement of cardiovascular diseases. It has been suggested that the gastrointestinal tract may be a privileged site for polyphenol cardioprotection [382], which would perform its action by prebiotic effect [377].

Mechanisms of action of polyphenols for cardiac health benefits include the reduction of plasma TG levels by increasing the activity of lipoprotein lipase (LPL), which decreases the concentrations of LDL-C in circulation [383]. Polyphenols also contribute to improving endothelial function and protection of the vessels by inhibiting LDL oxidation from reactive oxygen and nitrogen species [384]. They regulate the activity of nitric oxide synthase and the bioavailability of nitric oxide for the endothelium [385], mainly through the inhibition of C-dependent NADPH oxidase kinase protein and the inhibition of endothelin-1 vasoconstrictor. This, in turn, leads to reduced blood pressure and inhibition of platelet aggregation, secretion, and adhesion, which are responsible for atherosclerosis, stroke, or thrombosis [386,387]. 

The efficiency of the different factors in cardiovascular diseases is still debated. Several authors have demonstrated a cardioprotective effect of polyphenols in ischemic heart disease and heart failure [248,388,389]. However, the evidence is not conclusive [390,391]. Dietary intake of phytochemicals could have beneficial effects on the reduction of blood pressure, showing an inverse correlation with diastolic pressure [392] or both systolic and diastolic [393,394]. In contrast, other authors found no significant effects [395,396,397]. Among phytochemicals, flavanols and flavonols can prevent vascular lesions [398]; flavonoids in cocoa and soy showed favorable results on cardiovascular diseases [399], while others are not efficient [222]. Again, the consumption of coffee can have a protective effect thanks to its antioxidant properties, instead of a harmful effect due to the increase of the lipid fraction that damages the endothelium [400].

Vegetable consumption appears to be inversely related to the risk of cardiovascular diseases, attributable to bioactive components including vitamins, dietary fiber, proteins, and phytochemicals [401]. Different studies have shown that vegetables such as asparagus, celery, lettuce, broccoli, onions, tomatoes, potatoes, soybeans, and sesame have great potential in the prevention and treatment of cardiovascular diseases [401]. It has been reported that the total intake of fruit, regardless of the specific type of fruit (berries, pomegranates, grapes, citrus fruits, apples, strawberries), improves cardiovascular safety and leads to a reduction in CVD deaths, estimated at 6–7% for each portion of 80 g [402].

Results from the PREDIMED study showed a link between total polyphenol intake and the risk of cardiovascular-related events [248,280]. Evidence from meta-analyses of randomized controlled studies and observational studies demonstrated the beneficial effects of MEdD components, such as fruits, vegetables, legumes, nuts, and whole grains, in the prevention of cardio-metabolic risk [340,403,404]. The use of the MedD in subjects at high risk of cardiovascular diseases has shown an increase in non-enzymatic antioxidant capacity, an anti-inflammatory effect, a decrease in the biomarkers of atherosclerosis, and improvements in the lipid profile, insulin sensitivity, blood pressure, and carotid atherosclerosis [281,405].

The intake of MedD supplemented with EVOO has been shown to reduce the incidence of major cardiovascular events [406,407]. Phenolic compounds in olive oil prevent chronic inflammatory conditions [408,409,410] by reducing the expression of NF-KB and MAPK, which regulate the production and secretion of a variety of proinflammatory molecules [411,412]. Some studies [406] show that the integration of olive oil significantly improves flow-mediated dilation (FMD) [397,413], resulting in lowering the systolic and diastolic blood pressure in hypertensive individuals [413,414]. Among the polyphenols in olive oil, hydroxytyrosol (HT) has demonstrated proven anti-inflammatory and cardio-preventive effects [415], underscoring its importance in protecting LDL and consequently reducing the risk of cardiovascular diseases. HT has been described as the most effective inhibitor of inflammatory pathways that stimulate the production of NO, eicosanoid PGE2, and cytokines such as IL-1α, IL-1β, IL-6, IL-12, TNF-α, and gamma-induced interferon chemokines protein 10 monocyte chemoattractant protein 1 [416]. The European Food Safety Authority (EFSA) issued a guideline recognizing the efficacy of olive oil phenols (HT and its derivatives, 5 mg/day per 20 g olive oil) in protecting LDL from oxidation [417].

Epidemiological data and clinical studies suggest that the consumption of red wine is associated with a lower risk of CVD [418,419,420]. In individuals at high cardiovascular risk, the consumption of red wine (30 g alcohol /day) for 4 weeks leads to an increase in levels of Apo AI, Apo A2, and HDL [355]. The LDL/HDL ratio improves after a daily glass of red wine (0.1 L in women or 0.2 L in men) [421], both in people with carotid atherosclerosis and in subjects with dyslipidemia, after consuming red wine for 30 days (125 mL/d in women and 250 mL/d in men) [422]. 

Resveratrol can increase oxidative stress resistance through different signaling pathways, including SIRT1, nuclear factor erythroid-related factor 2, and nuclear factor κB [423]. SIRT1 influences gene expression and physiological homeostasis, exerting extensive metabolic effects [424]. Considering that inflammation plays a role in atherogenesis [363], resveratrol inhibits the activity of inflammatory enzymes such as cyclooxygenase and lipoxygenase [425], interleukin production such as IL-1, IL-2, IL-12, IL-6, IL-8, IFN-ɣ, TNF-α [426,427,428,429], and attenuates proinflammatory transcription factors and protein-1 activator [430]. Therefore, the multitude of resveratrol effects is related to interactions with numerous molecular targets [431,432]. However, the resveratrol paradox (low bioavailability and high bioactivity) still raises undefined doubts [433,434]. 

Several studies have investigated the timing, dosage, bioavailability, and toxicity of resveratrol, particularly in the context of diabetes, obesity, cardiovascular disease, cancer, and neurodegenerative disorders [435]. These studies have highlighted the challenges in defining the safety/effectiveness of resveratrol doses for different populations. Conflicting information has been reported, underscoring the need for further research before recommending the widespread use of resveratrol [436]. A recent meta-analysis study on risk thresholds for alcohol consumption found that the threshold for the lowest risk of all causes of mortality was around 100 g per week. However, concerning different types of CVD, the consumption of 100 g per week of alcohol showed a harmful association [437]. 

The effectiveness of the different polyphenols, concerning their specific mechanisms of action on cardiovascular diseases, remains debated. The cardiovascular benefits of polyphenols from plants can be influenced by the amount and the mutual interactions between polyphenolic compounds, which work through distinct pathways to achieve synergistic action [438].

#### 3.1.4. Neuroprotective Effect of Polyphenols

A multidisciplinary approach, incorporating diet, physical exercise, and cognitive training, has been shown to enhance or preserve cognitive function [439]. Oxidative stress and damage to brain macromolecules play crucial roles in the development of neurodegenerative diseases [440]. It is hypothesized that the antioxidant properties of many polyphenols can provide neuroprotection. Therefore, controlling neurodegenerative diseases involves balancing the generation of ROS and their elimination by antioxidants. Pre-clinical studies have shown promising results; however, the beneficial effects of antioxidant therapy for neurodegenerative diseases remain a subject of debate [441,442]. For example, dietary saffron may be considered suitable for neuroprotection due to its low cytotoxicity and its ability to cross the blood-brain barrier [443]. Stem-cell therapy seems to be the only hope for neurological reconstruction in cases of severe nerve damage and neurodegenerative disorders [444].

Brain function and the gut microbiota are linked [445,446]. Alterations in this axis can affect many diseases in humans [447] and influence mood, depression, anxiety [448], and cognition in Alzheimer’s disease [449]. Among the extrinsic factors able to modulate this connection are reported mainly the components of the diet, such as polyphenols, which perform neural protection due to their antioxidant and anti-inflammatory properties. They act both directly in the brain for the ability to cross the blood-brain barrier and indirectly through the modulation of the microbiota and the gut-brain axis [450], suggesting that the combination of prebiotics, MedD, and exercise can relieve and counteract neurodegenerative diseases by regulating the intestinal microbiota.

The positive impact of flavonoid-rich products in diets has been associated with the prevention of neurodegenerative disorders and the improvement of cognitive functions [451,452]. Flavonoids demonstrate the ability to scavenge free radicals and promote neuronal survival in the hippocampus [265]. The consumption of fruit and vegetable juices, particularly those containing polyphenols from grapes, has been associated with delaying the onset and progression of Alzheimer’s disease (AD) [453,454,455]. Moderate consumption of red wine (rich in stilbenes) has been shown to reduce the progression of β-amyloid, thereby attenuating cognitive deterioration and the prevalence of AD [456]. Clinical and epidemiological studies suggest that the consumption of flavonoids and polyphenols, including compounds such as catechin, resveratrol, and curcumin from fruits, vegetables, or beverages [457], along with increased adherence to a MedD, may reduce the risk of AD [458].

Different studies have reported that adherence to MedD has a protective effect against depression [459,460]. Fruits and vegetables, rich in antioxidants such as β-carotene, tocopherols, ascorbic acid, polyphenols, and anthocyanins, play a crucial role in reducing oxidative stress and neural damage [461,462]. Fish and nuts, sources of long-chain omega-3 polyunsaturated fatty acids, contribute to modifying cell membrane structure and function, influencing cellular communications, reducing inflammatory processes, and enhancing neurotransmitter activities [463]. Additionally, limiting the consumption of red and processed meats, which are associated with inflammation and depression [464,465], can contribute to reducing the occurrence of depression. However, further studies are needed to reach a definitive conclusion regarding the effects of the MedD on depression. Precisely defining the beneficial effects of polyphenols on neuroprotection remains challenging due to the multitude of polyphenolic compounds and their metabolites in fruits, vegetables, and drinks, coupled with their variable bioavailability linked to individual consumers and intraindividual responses in pathophysiological conditions. 

#### 3.1.5. Challenges of Research on Polyphenols

Several studies have demonstrated the beneficial effects of plant-based foods in preventing chronic diseases such as T2D, obesity, CVD, and neurological disorders [340,443,466]. It has been highlighted that polyphenols can oxidize free radicals and prevent injuries caused by them through their direct radical-scavenging ability [467] and by reducing their pro-oxidant activity [468], including chelating metal ions [469]. However, other studies [470,471] have not consistently shown the effectiveness of fruits and vegetables in producing health effects or preventing chronic diseases. This suggests the presence of subtypes with different nutrient content within large food categories, and these subtypes may be differentially associated with negative health consequences [472]. Additionally, the concentration of phenolic compounds present in plants could vary significantly in the human diet due to the preparation and processing of food (e.g., cooking, frying, freezing), which plays a crucial role in determining their optimal dose in a diet [473].

The current challenge regarding the use of polyphenols as clinical agents for health concerns their low oral bioavailability [23,223,474]. Many efforts are underway to enhance their bioavailability, such as micro- and nanoencapsulation, showing promising results [475,476,477]. In vitro, animal, and human studies have shown the strong metabolization and transformation of polyphenols, leading to the formation of metabolites with higher bioactivity than the parent molecules [229,230]. In addition, factors such as methylation, glucuronidation, sulfation, and co-digestion of other phytochemicals can alter the bioavailability of phenolic compounds in the human body [478]. Polyphenolic metabolites, produced by the liver, small intestine, and intestinal microbiota, play a considerable biological role. However, further research is needed to deepen and define the relationships between the bioavailability and bioactivity of polyphenols and their metabolites that can influence health outcomes. The interaction of polyphenols with other components in diets and the intestinal microbiota must also be investigated [479,480]. The synergistic and antagonistic activities of polyphenols concern other biological antioxidants in the body’s defence. For example, ascorbate and catechin have a synergistic effect since ascorbate could protect catechin from oxidation [481]. 

Furthermore, the ability of polyphenols to cross the blood-brain barrier to exert their protective effects is not always adequate [482]. Another problem concerns the relationship between phenolic compounds’ dose and effect benefits [483] and their potential side effects of long-term exposure in humans [473]. Again, phenolic compounds can show pro-oxidant activity, raising concerns about their consumption. While they may exhibit antioxidant properties in vitro [316,484] they can act as pro-oxidants in vivo [485]. Moreover, excessive consumption may change the activity of endogenous antioxidants [196]. 

Polyphenols may constitute a new pharmacological approach to managing MS, but further studies are needed for the formulation of new, suitable, and safe compounds. While most polyphenol studies are conducted in vitro and in vivo on cell cultures or animal models, clinical studies are scarce. 

It is necessary to emphasize that the beneficial effects of polyphenols can occur only through frequent and long-term intake as part of a healthy and diversified diet [438]. In addition, the complex nature of MS involves many interconnected factors such as obesity, insulin resistance, hypertension, and dyslipidemia, while polyphenolic compounds have different mechanisms of action and may have different effects on these components [486], which may result in a lack of uniform association with MS. For example, some polyphenols may have a greater impact on blood glucose levels, while others may be more effective in reducing body weight or blood pressure [487]. Thus, the association between polyphenols and MS may not be universally observed and may vary depending on the specific polyphenolic compounds and their interactions with the complex metabolic pathways involved in MS.

It is necessary to carry out clinical studies on the concentration, bioavailability, pharmacological, and toxicological evaluation of phenolic compounds. Additionally, research on the metabolism and biological activity of phenolic compounds in the human body is crucial.

## 4. Beneficial Effects of Physical Activity on Metabolic Syndrome

**Figure 3:**
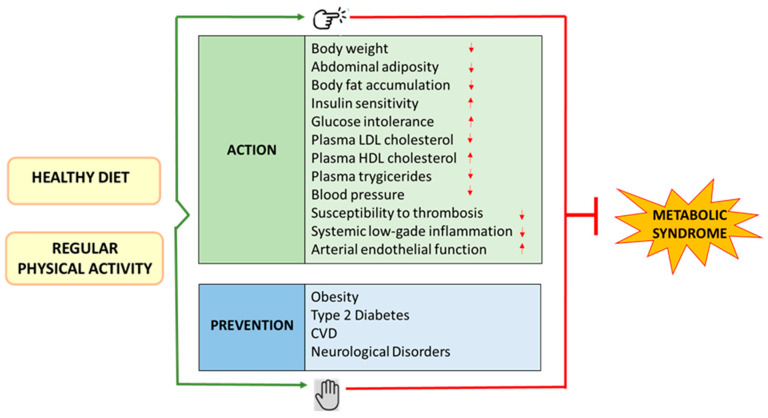
Strategic approaches to prevent and treat Metabolic syndrome.

Lifestyle is important in the prevention and treatment of obesity, diabetes, and diseases linked to MS. The managing risk factors linked to MS suggest lifestyle changes, including modifications to diet, by using appropriate nutrients and foods containing polyphenols in combination with moderately regular physical activity (Figure 3).

A sedentary lifestyle characterizes all age groups. Boys between 6 and 17 years old perform less than half of the recommended exercise quota. Between the ages 12 and 21, about half of men and two-thirds of women do not regularly perform physical activity. Only 22% of adults have regular physical activity [488]. Lack of physical activity is the fourth risk factor for mortality [489] and leads to a 30% increase in mortality compared to people exercising 30 min per day [490]. The recent Global Action Plan on Physical Activity (2018–2030) set a target of a 10% reduction in physical inactivity levels by 2025 in all countries.

Exercise is characterized by every body movement caused by skeletal muscles that require energy expenditure [491]. Exercise typically includes aerobic training, which affects cardiovascular fitness [492], and endurance training, which aims at muscle mass and strength [493]. The benefits obtained are related to the quantity and intensity of the exercise [494,495]. However, a small amount of exercise, even if below the recommended levels, can improve the quality of daily life [495]. Reasonable exercise can increase energy expenditure, strengthen muscles, reduce blood pressure and blood lipids, increase bone mass, and regulate psychological processes [496,497,498]. Several studies indicate that changing lifestyles [499] with an increase in physical exercises [500], weight loss [501,502], and adherence to a healthy diet [461] have beneficial effects and can result in the reversal of MS and its constituents. 

**Figure 4:**
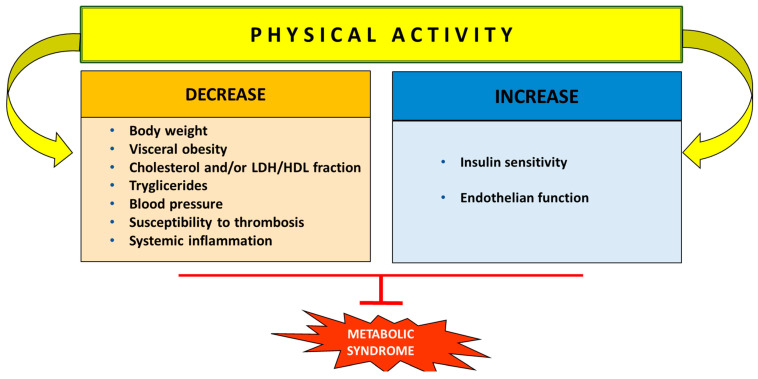
Beneficial effects of physical activity in the prevention of metabolic syndrome and associated diseases.

Physical activity has beneficial effects on MS (Figure 4). Weight loss is among the most important strategies to manage the risk of co-morbidity from obesity, such as diabetes, cardiovascular, pro-thrombotic, and inflammatory risks of MS [503]. Exercise reduces fat mass in overweight/obese people [504,505,506,507]. In addition, physical exercise improves glucose and lipid metabolism, reduces plasma levels of triglycerides, and increases HDL cholesterol, lowering circulating adipokines such as resistin, visfatin, and fatty-acid binding protein IL-6, which are involved in the development of metabolic disorders and inflammation [508,509]. Epidemiological studies indicate that weight loss can improve the sensitivity and action of insulin and reduce the risk of developing T2D [509]. An increase of 20% in the risk of diabetes has been estimated for each 2-h daily increase in watching television [510]. 

Physical activity is associated with a significant reduction in the risk of T2D [511]. Benefits of exercise include increased insulin sensitivity, improved glycaemic control, and prevention of cardiovascular disease [512,513]. Several studies have highlighted reducing, delaying, and reversing insulin resistance and T2D [514]. Routine exercise along with a controlled dietary intake (reduced fat, increased fiber, and frequent consumption of fruit, vegetables, etc.) can be the first course of action for the prevention and clinical treatment of T2D.

Lifestyle interventions that increase physical activity and reduce calorie intake have a positive impact on metabolic outcomes. A healthy diet associated with adequate physical activity can have greater effects than diet alone or physical exercise [515,516,517]. When obese subjects are deprived of 500 kcal daily, the use of aerobic and endurance exercises has improved their MS response compared to diet alone, particularly in terms of reducing waist circumference, body fat percentage, waist-to-hip ratio, fasting blood glucose level, triglyceride level, total cholesterol, low-density lipoprotein cholesterol, and very low-density lipoprotein cholesterol (VLDL-c) [517]. Lee et al. [518] reported that both aerobic and resistance exercise performed for 180 min per week improved cardiorespiratory fitness and reduced total fat, visceral adiposity, waist circumference, and intrahepatic lipids in adolescent boys with obesity. Regular and reasonable exercise is a key factor in improving blood glucose control, insulin sensitivity, lipid profile, blood pressure, body composition, and fitness [519,520,521].

A diet-exercise regime counteracts oxidative stress and MS. Then, peroxisome proliferator-activated receptor gamma coactivator-1 alpha (PGC-1α) promotes mitochondrial biogenesis and the increase of respiratory enzymes, leading to the improvement of mitochondrial and lipid metabolism [522,523] as a target for the prevention or treatment of MS and various diseases such as diabetes and CVD [523,524,525]. The skeletal muscle metabolic function accounts for about 80% of postprandial glucose disposal [526], due to the ability of exercise to improve the energy consumption of the muscles. 

Exercise/muscle contraction increases ROS production and promotes oxidative stress [527], which is necessary for glucose uptake [528]. During contraction, H_2_O_2_ increases glucose absorption [529], while N-acetylcysteine reduces glucose absorption induced by contraction [530]. Exercise/muscle contraction can lead to glucose uptake, insulin-independent, through various molecules such as AMP-activated kinase (AMPK), Ca^2+^ calmodulin-independent protein kinases, hepatic kinase B-1, and protein kinase C [531,532], which facilitate the translocation of GLUT4 to the plasma membrane, leading to increased glucose uptake. In addition, exercise increases the uptake of fatty acids from circulation into muscle cells [533] and further from the cytosol into mitochondria [534]. Endurance exercise has shown benefits, especially in diabetes. It improves insulin sensitivity and glucose tolerance and facilitates weight management [535].

The combination of both types of exercises, aerobic and endurance, reduces the risk of developing coronary heart disease, stroke, and T2D [536]. Exercise has beneficial effects on coronary microvascular and left ventricular function in individuals with obesity [106,537]. Several mechanisms have been proposed to explain the beneficial effect of exercise on coronary heart disease, such as heart preconditioning, regression of plaque formation, and increased coronary artery collateral [538,539]. In individuals with physical activity, cardiac preconditioning reduces heart attack damage (ischemic/reperfusion) by 30–40% [539]. Exercise protects against heart attack through increased clearance of ROS [540], such as the enzyme manganese superoxide dismutase (MnSOD), mainly found in the mitochondria [539]. A beneficial action on arteriosclerosis occurs through coronary collateral involving arterioles, which can reduce the size of the heart attack by increasing the retrograde blood flow to the ischemic myocardium [541,542].

Plaque accumulation can result in coronary stenosis and myocardial infarction. Beneficial effects of exercise on plaque regression were reported [543] through various mechanisms, such as the increase in HDL and the reduction of LDL, along with the elimination of macrophages and foamy cells from the necrotic nucleus of the lesion [544]. Aerobic training affects cardiovascular fitness [492]. Aerobic exercise affects the vessels, heart, and muscles. In particular, for myocardial function, the progressive workload participates in the remodeling of the heart with a progressive increase in VO_2_ max, associated with a lower cardiovascular risk. It is recommended to walk and cycle 3 to 5 times a week, as they involve multiple muscle groups.

Therapeutic approaches to neuroprotection in the context of MS involve a dietary lifestyle and physical exercise through a multidisciplinary approach in order to prevent or limit the progression of neuronal degeneration. Exercise is crucial in reducing the risk of cognitive function decline and dementia [545,546]. The exact mechanisms by which physical activity affects cognitive processes are not fully defined [547,548]. The beneficial effects of exercise are attributed to the increased bioavailability of neurotrophins such as brain-derived neurotrophic factor (BDNF) and vascular endothelial growth factor (VEGF) through aerobic training, and insulin-like growth factor1 (IGF-1) through resistance training. 

Aerobic training [549] and endurance [550] improve cognitive function and the integrity of the white matter [551]. Exercise increases cerebral blood flow and the bioavailability of nitric oxide, delaying arterial stiffening [552]. Thus, endothelial function is improved, which is important for the regeneration of cerebral white matter [553,554], and vascular risk factors such as hypertension, diabetes mellitus, and hypercholesterolemia, associated with subcortical ischemic vascular cognitive impairment, are reduced [555]. 

It has been reported that running therapy has beneficial antidepressant effects [556] by acting on monoaminergic mechanisms and cerebral blood flow [557]. Moreover, it has been suggested that a ten-year reduction of 10–20% in the risk factors of dementia in old age—poor education, middle-aged hypertension, middle-aged obesity, diabetes, physical inactivity, smoking, and depression [558]—could reduce by 8–50% the worldwide prevalence of AD in 2050 [559].

## 5. Conclusions

In conclusion, this paper has elucidated the intricate interplay between nutrition and physical activity in managing MS, with profound implications for combating diabesity, improving cardiovascular health, and potentially mitigating neurodegenerative diseases. Therapeutic strategies suggested include integrative approaches aimed at improving lifestyle and daily routine, such as diet and physical activity. MedD, as a high-quality diet, has protective effects against MS, partly attributed to polyphenols, most commonly found in plant-based food sources such as vegetables, cereals, spices, olive oil, nuts, fruits, and beverages. A combination of phytochemicals, rather than a single dietary polyphenol, is suggested. However, more research is needed to better understand and define the relationships between the bioavailability and bioactivity of polyphenols and their metabolites that can influence health outcomes, as well as the real value of dietary polyphenols in preventing the progression of MS and related diseases. Modulation of the gut microbiota reduces the risks associated with MS, improves diabesity and CVD, and exerts neuroprotective action. Lifestyle interventions that involve physical activity and reducing calorie intake can improve metabolic outcomes and MS. Combining a slight daily calorie restriction (500 kcal) with aerobic and endurance exercises can significantly improve the response to MS compared to diet alone [517]. Thus, a complete and balanced diet, coupled with regular and reasonable exercise, can be key factors in preventing MS, cardiovascular, and neurodegenerative disorders. As such, healthcare practitioners should emphasize the importance of personalized lifestyle interventions tailored to individual needs, promoting sustainable behavior changes that empower individuals to take charge of their metabolic health and overall well-being.

## Data Availability

Not applicable.
